# Secure Messaging Intervention in Patients Starting New Antidepressant to Promote Adherence: Pilot Randomized Controlled Trial

**DOI:** 10.2196/51277

**Published:** 2023-12-08

**Authors:** Carolyn Turvey, Lindsey Fuhrmeister, Dawn Klein, Kimberly McCoy, Jane Moeckli, Kenda R Stewart Steffensmeier, Natalie Suiter, Jen Van Tiem

**Affiliations:** 1 Center for Access and Delivery Research and Evaluation Iowa City VA Health Care System Iowa City, IA United States; 2 Office Rural Health Veterans Rural Health Resource Center Iowa City, IA United States; 3 Department of Psychiatry Carver College of Medicine University of Iowa Iowa City, IA United States; 4 JP Systems Clifton, VA United States

**Keywords:** depression, text messaging, medication adherence, medication, medications, adherence, antidepressant, antidepressants, depressive, text message, text messages, messaging, SMS, veteran, veterans, military, randomized controlled trial, RCT, controlled trials, mental health, psychiatry, mobile phone

## Abstract

**Background:**

There are a range of effective pharmacological and behavioral treatments for depression. However, approximately one-third of patients discontinue antidepressants within the first month of treatment and 44% discontinue them by the third month of treatment. The major reasons reported for discontinuation were side effect burden, patients experiencing that the medications were not working, and patients wanting to resolve their depression without using medication.

**Objective:**

This study tested the acceptability, feasibility, and preliminary effectiveness of an SMS messaging intervention designed to improve antidepressant adherence and depression outcomes in veterans. The intervention specifically targeted the key reasons for antidepressant discontinuation. For example, the secure message included reminders that it can take up to 6 weeks for an antidepressant to work, or prompts to call their provider should the side effect burden become significant.

**Methods:**

This pilot was a 3-armed randomized controlled trial of 53 veterans undergoing depression treatment at the Iowa City Veterans Affairs Health Care System. Veterans starting a new antidepressant were randomized to secure messaging only (SM-Only), secure messaging with coaching (SM+Coach), or attention control (AC) groups. The intervention lasted 12 weeks with follow-up assessments of key outcomes at 6 and 12-weeks. This included a measure of antidepressant adherence, depressive symptom severity, and side effect burden.

**Results:**

The 2 active interventions (SM-Only and SM+Coach) demonstrated small to moderate effect sizes (ESs) in improving antidepressant adherence and reducing side effect burden. They did not appear to reduce the depressive symptom burden any more than in the AC arm. Veteran participants in the SM arms demonstrated improved medication adherence from baseline to 12 weeks on the Medication Adherence Rating Scale compared with those in the AC arm, who had a decline in adherence (SM-Only: ES=0.09; *P*=.19; SM+Coach: ES=0.85; *P*=.002). Depression scores on the 9-Item Patient Health Questionnaire decreased for all 3 treatment arms, although the decline was slightly larger for the SM-Only (ES=0.32) and the SM+Coach (ES=0.24) arms when compared with the AC arm. The 2 intervention arms indicated a decrease in side effects on the Frequency, Intensity, and Burden of Side Effects Ratings, whereas the side effect burden for the AC arm increased. These differences indicated moderate ES (SM-Only vs AC: ES=0.40; *P*=.07; SM+Coach: ES=0.54; *P*=.07).

**Conclusions:**

A secure messaging program targeting specific reasons for antidepressant discontinuation had small-to-moderate ES in improving medication adherence. Consistent with prior research, the intervention that included brief synchronic meetings with a coach appeared to have a greater benefit than the SMS-alone intervention. Veterans consistently engaged with the SMS messaging in both treatment arms throughout the study period. They additionally provided feedback on which texts were most helpful, tending to prefer messages providing overall encouragement rather than specific wellness recommendations.

**Trial Registration:**

ClinicalTrials.gov NCT03930849; https://clinicaltrials.gov/study/NCT03930849

## Introduction

### Background

The lifetime prevalence of major depression in the United States ranges from 13% to 17% [1]. The Global Burden of Disease study places major depression within the top 3 sources of disability-adjusted life years in the United States and Europe [2]. There are a range of effective pharmacological and behavioral treatments for depression [3]. However, many patients and health care providers must work through multiple trials before finding the optimal regimen. The Sequenced Treatment Alternatives to Relieve Depression trial found that 37% of the patients responded to the first antidepressant prescribed, but up to 4 medication changes were needed to achieve a 67% response rate overall [3-5].

Patient adherence to depression medication treatment contributes significantly to these treatment difficulties. Approximately one-third of patients discontinue antidepressants within the first month of treatment and 44% discontinue them by the third month of treatment [6]. Kales et al [7] examined veterans recently prescribed antidepressants and found 29% nonadherence based on self-report and 53% nonadherence when based on the medication possession ratio using veterans Affairs (VA) pharmacy data. Samples and Mojtabai [8] conducted a national survey and found that the major reasons reported for discontinuation were side effect burden, patient experience that the medications are not working, and patients wanting to resolve depression without using medication. Although many patients attribute nonadherence to simple forgetting, Gadkari and McHorney [9] demonstrated that negative beliefs about medication predict unintentional nonadherence (eg, forgetting doses and running out of medication).

To improve antidepressant adherence, providers need effective tools to address reasons for self-discontinuation [8]. There have been many commercial mobile apps or texting technologies developed to promote medication adherence and management across a range of medical conditions [10]. Meta-analytic reviews of the use of mobile apps to promote psychiatric medication adherence in community settings indicate small to moderate effect sizes (ESs) [11-13]. One observed limitation of mobile app use in health care has been a significant drop off in use and patient engagement with the application over time [11]. Much of this prior work examined general apps that were not tailored to a specific medication (eg, antidepressants), and many were not integrated into the overall treatment setting, which may be the critical factor. Mohr et al [14] in an article entitled, “Three Problems with Current Digital Mental Health Research...and Three Things We Can Do About Them,” argue that mobile apps’ limited effectiveness is because of their being developed and tested outside of the clinic setting where they can have the most impact.

### Objectives

This study tested the acceptability, feasibility, and preliminary effectiveness of an intervention designed to improve adherence and depression outcomes among veterans initiating antidepressant medication. In the intervention, veterans used Annie, the Department of VA mobile app SMS, to support self-management of antidepressant medication use. Implementation and adoption of Annie have been explored in self-support for the hepatitis C virus. This pilot indicated low-to-moderate adoption and perceived value of the messaging service, but among adopters, there was enhanced treatment adherence and lower distress about failing hepatitis C virus treatment [15]. Shimada et al [16] reported that secure messaging adoption in VA was enhanced by leadership and technical support at the facility level, and early adopters of secure messaging in primary care demonstrated lower urgent care use.

For the program described herein, we purposely designed the Annie protocol to counter the key reasons for antidepressant self-discontinuation, such as side effect burden or prolonged delay (typically 6 wk) of treatment effect. To increase Veteran and provider satisfaction with Annie, study staff provided participants and providers visual representations of their Annie responses (ie, graphs and bar charts) between medical visits. Veterans also received training about My HealtheVet (MHV), VA’s patient portal, as a communication tool should any medication-related issues arise.

## Methods

### Recruitment

This pilot was a 3-armed randomized controlled trial of 53 veterans undergoing depression treatment at the Iowa City VA Health Care System. The proposed protocol was shared with the Iowa City VA Veteran Engagement Panel where veterans provided feedback and recommendations based on patient experiences. The panel members introduced the idea of presenting visual representations of patient-centered outcomes, such as side effect burden, in addition to the standard use of graphs to chart depressive symptom severity over time. The panel members also recommended that Annie’s responses include information about the Veteran Crisis Line and use more encouraging and patient-centered language for responses related to veterans experiencing medication side effects.

### Ethical Considerations

All aspects of the protocol were approved by the University of Iowa and Veteran’s Administration internal review board (IRB ID#201712732). It is registered on ClinicalTrials.gov (NCT03930849). The study coordinator provided informed consent to all participants, clearly describing the 3 arms of the study, the nature of the randomization, the amount of time involved, and all potential benefits and harms of participation. All study data were deidentified for the preparation of this manuscript. Participants in all treatment arms were reimbursed US $25 for completing all assessments for the study.

### Inclusion and Exclusion Criteria

Veterans were eligible for the study if they were seeking care at either the Iowa City VA Health Care System Primary Care or Mental Health Service Line and were initiating new antidepressant treatment for depression OR they had a recent change in antidepressant medication. New antidepressant treatment was defined as the initiation of antidepressant treatment and no indication of any antidepressant use in the previous year. Veterans with medical record indications of comorbid major psychotic disorders, current active substance abuse, or cognitive disorders were excluded. Those with comorbid posttraumatic stress disorder were eligible. Veterans were not required to be experienced in using texting. However, they did need to own a cell phone (flip phone, smartphone, or Android) with texting functionality and a data plan that allowed for texting.

### Enrollment

Veterans who met initial eligibility criteria were mailed an invitation letter that described the study and contained information on how to contact staff if they had questions or wanted to express whether they were interested in participating. Study staff then followed up with a phone call to veterans. Those who were interested and eligible were scheduled for an in-person visit with a study coordinator at a location convenient for them, either at their local VA clinic or at their home. At the visit, veterans went through consent procedures, were randomized to 1 of the 3 arms, as described in the *Data Collection and Intervention Descriptions* section, and then completed baseline assessments. Randomization was blocked by recruitment within the primary care or Mental Health Service Line at a local VA medical center. We recruited 8 veterans from primary care and 45 veterans from the Mental Health Service Line. The baseline assessments included the assessments described in the *Data Collection and Intervention Descriptions* section. The primary outcome was a change in self-reported medication adherence. The secondary outcomes were changes in depressive symptom burden and side effect burden.

### Data Collection and Arm Descriptions

#### Secure Messaging Only and Secure Messaging With Coaching Intervention Arms

After the visit, the study staff completed the Annie program registration for the Veteran participant and they began receiving texts from Annie. Both interventions (secure messaging only [SM-Only] and secure messaging with coaching [SM+Coach]) received the same SMS text messaging protocol (Table 1). The prompts were designed to specifically target key causes for antidepressant nonadherence. Veteran participants were asked about perceived effectiveness, side effect burden, adherence, and depressed mood. Their response to this question determined Annie’s subsequent response. For example, if a Veteran participant reported that they did not believe the antidepressant was working, they received a text reminding them that it could take up to 6 weeks for the medication to impact their mood. If experiencing significant side effects was endorsed, Annie recommended that they contact their provider for discussion. In addition, Annie asked questions about engaging in meaningful activities and progressing toward therapeutic goals. In developing Annie’s script, we generated multiple possible responses for many of the Veteran participant prompts to prevent tedious repetitiveness in Annie’s interactions.

To reduce question burden, Veteran participants responded to 2 questions each day, although the content of the questions rotated and followed the specific order indicated in Table 1. After responding to the daily questions, Annie provided a quote or tip to encourage or motivate them. They were then asked to rate on a scale from 1 to 10 the degree to which they found the quote or tip helpful. These quotes and tips rotated throughout the 12-week intervention period and are shown in the *Results* section.

The study staff monitored the Veteran participant’s medical record to determine the date and time of the next follow-up appointment tied to the management of the antidepressant medication. The standard practice in both the Iowa City VA Mental Health and Primary Care clinic is to schedule a 4- to 6-week follow-up appointment after initiating a new antidepressant and a 12-week follow-up to ensure continued benefit. Although there was some variability in the degree to which clinics and veterans had 2 appointments within the 12-week period, the majority had at least one follow-up scheduled within the proposed study period.

Each week, the study staff extracted Annie’s response data indicating depressive symptom severity, antidepressant adherence, side effect burden, and functioning and displayed the trajectory of each outcome on a color-coded graph. These graphs were shared with the Veteran participant via MHV and postal mail. Before their follow-up medical appointment for antidepressant management, they were reminded of the appointment and asked to take their most recent Annie Response Graph for discussion with their provider. Their provider also received a copy of the most recent graph via secure messaging.

**Table 1:** The Annie texting protocol veterans received over 12 weeks.

Question and day of week administered			Possible veteran responses		Example of Annie’s response tethered to Veteran response
**Hi, It’s Annie. Please rate your current mood with 1 being the worst mood and 10 being the best mood. Text MOOD and your response like this: Mood 6**					
	Monday, Wednesday, Friday	1-4		Sorry to hear your mood is low today. Remember you can call the Veterans Crisis Line: 1-800-273-8255 if you need to talk to someone.	
	Monday, Wednesday, Friday	5-6		Thanks for the feedback- remember to take your medicine.	
	Monday, Wednesday, Friday	7-10		That is good to know. Continue to take your medicine and let me know how your mood is next time.	
**Have you missed any medication doses in the past week? Yes or No. Please text MISSED and your response like this MISSED no. Thanks, Annie**					
	Friday	Yes		It is important to take your medications as prescribed. Please call your treatment team if you are not taking your medication as recommended.	
	Friday	No		Taking your medications as prescribed helps you get the most benefit from them	
**Do you feel your medication is working? YES, NO, or UNSURE. Please text WORKING and your response like this: Working yes. Thanks, Annie**					
	Monday	Yes		Great to hear that the medicine is doing its job. Keep taking your medication.	
	Monday	No or unsure responses		Thanks. Remember, most people need to take this type of medication regularly for four to six weeks to begin to get the full effect.	
	Monday			Thanks for responding. It might take some more time for the medicine to start working.	
	Monday			Did you know it can take up to 2 months for this kind of medicine work? Taking your medicine will help to figure out if this is the right treatment.	
**Annie here. Are you experiencing side effects from your medication? NONE, MILD, MODERATE or SEVERE. Text EFFECTS and your response like this: Effects mild**					
	Wednesday	None		Good to hear you are not having problems. Keep taking your medication as prescribed.	
	Wednesday	Mild		Thanks for answering. Many people taking medications have at least one side effect. Contact your treatment team if you have questions.	
	Wednesday	Mild		Thanks for answering. I am sorry to hear that. Contact your treatment team if you have questions.	
	Wednesday	Mild		Thanks for answering. If these side effects are bothering you may want to call your treatment team.	
	Wednesday	Moderate		Thanks for answering. Side effects may occur with a change in medication. If you are concerned, contact your provider to discuss these problems.	
	Wednesday	Moderate		Thanks for answering. Many side effects will go away after a few weeks. You may want to talk with your treatment team about any side effects.	
	Wednesday	Severe		Thanks for answering. Side effects may occur with a change in medication. Please contact your provider to discuss these problems.	
	Wednesday	Severe		Thanks for answering. If you feel that you are having severe side effects contact your treatment team so they can help you.	
**How often have you done enjoyable or meaningful activities? No Days, Some Days, Most Days, Every Day? Text ENJOY and your response: ENJOY Some days. Annie**					
	Tuesday, Thursday	No Days		Sorry to hear that. See if you can try to do one activity you enjoy.	
	Tuesday, Thursday	Some Days, Most Days, Every Day		Thanks. Hope you have a good day.	
**How much progress are you making toward your personal goal? NONE, A LITTLE, SOME, A LOT. Text MYGOALS and your response like this: Goal Some. Annie**					
	Tuesday, Thursday	None		Thanks. Sorry to hear no progress. Still stick with your treatment plan and report next time.	
	Tuesday, Thursday	None		Thanks. Sorry to hear you are having a tough time achieving your goal. Try setting a smaller goal for now.	
	Tuesday, Thursday	A little		Thanks. Great to hear you are making progress. Keep it up.	
	Tuesday, Thursday	A little		Thanks. A little progress is great. Keep working toward your goal.	
	Tuesday, Thursday	Some		Thanks. Great news. Have a good day.	
	Tuesday, Thursday	Some		Thanks. Good to hear you are making some progress on your goals.	
	Tuesday, Thursday	Some		Thanks. Awesome. Keep doing what you are doing to reach your goal.	
	Tuesday, Thursday	A lot		Thanks. Great news. Have a good day.	
	Tuesday, Thursday	A lot		Thanks. Good to hear you have made a lot of progress.	
	Tuesday, Thursday	A lot		Thanks. Fantastic. Keep up the good work.	

#### SM+Coach Arm

In addition to what is described above, Veteran participants randomized to the SM+Coach arm received a weekly coaching call with a study staff member. Together, they reviewed the Veteran participant’s weekly data reported via Annie and the current Annie Response Graph, looking at trends over time. During these coaching calls, they were also asked to reflect on the trends and identify any self-management issues that were emerging.

#### Attention Control Arm

The attention control (AC) arm was included to control for nonspecific therapeutic factors such as coach attention or using a new technology. This would ensure that any benefits observed were because of the specific intervention components described above. Following consent and randomization, the staff reviewed with the Veteran participant the importance of taking medications as prescribed, common logistic and attitudinal barriers to adherence to antidepressant treatment, expected timeline for response to antidepressant treatment, and some of the common side effects associated with these medications. They were also provided educational materials about MHV and offered assistance for registration if requested. In this arm, staff contacted Veteran participants just before their follow-up medical appointment for antidepressant management occurring within the 12-week follow-up period to discuss any general concerns they may be experiencing. If any issues were raised that required more clinical knowledge, the study staff encouraged them to address these issues with their provider. There were no other study contacts in this arm other than assessments of primary study outcomes, as described below.

#### Study Assessments

Although the intervention arms entailed multiple weekly assessments, the main study assessments were independent of Annie’s scripted assessments of depressed mood, as the AC arm did not receive Annie’s messages. The study quantitative assessments were performed at baseline and 6 and 12 weeks after baseline. This is the standard follow-up timeframe for evaluating the effectiveness of an antidepressant after it is initiated. Qualitative interviews for Veteran participants were conducted after they completed their final quantitative assessments. The results from these qualitative interviews are reported in a prior publication [17]. Quantitative assessments were mailed at weeks 6 and 12 after baseline.

The primary quantitative outcome of this study was antidepressant medication adherence using the Medication Adherence Rating Scale (MARS) [18]. In addition, we assessed secondary outcomes, depression symptom severity using the 9-Item Patient Health Questionnaire [19], and side effect burden using the Frequency, Intensity, and Burden of Side Effects Ratings [20]. Process measures were collected throughout the study to assess how Veteran participants were using Annie.

### Statistical Analysis

The primary hypothesis was that Veteran participants in the SM+Coach and the SM-Only interventions would have better self-reported antidepressant treatment adherence than those in the AC condition as indicated by the total MARS score measured at baseline and 6 and 12 weeks after baseline. As this was a pilot study with a small sample size, we conducted a mixed effects model including a group (SM-Only, SM+Coach, and AC) by time (baseline, 6 wk, and 12 wk) interaction term to derive an *F* value and significance level for type 3 fixed effects. ESs were calculated separately for each intervention by comparing SM-Only versus AC and SM+Coach versus AC. We did so by determining an ES metric where the difference between the mean change scores for the 2 being compared was the numerator. The denominator for this metric was the SD of the combined mean change scores for the 2 intervention arms being compared [21]. ESs were categorized where 0.2−0.5=small ES, 0.5−0.8=moderate ES, and ≥0.8=large ES [22].

## Results

### Overview

For this pilot study, 327 veterans were approached to participate. Of these, 120 (36.7%) were not interested, 72 (22%) were unable to contact, and 23 (7%) were ineligible. Of the 53 patients who provided consent, 3 dropped out before starting the study. Sample characteristics are presented in Table 2. The average age was 46 (SD 13.62) years, with 28% (15/53) women, 4% (2/53) African American, and 8% (4/53) Hispanic. Initial sample sizes immediately after randomization were 17 for the SM-Only arm, 18 for the SM+Coach arm, and 18 for the AC arm. Throughout the 12-week intervention, 4 discontinued SM-Only, 4 discontinued SM+Coach, and none discontinued AC. For each of the 3 arms, the sample was predominantly male, consistent with the Veteran enrollee population as a whole.

**Table 2:** Veteran characteristics and outcome measures results (N=53).

		SM-Only^a^ (n=17^b^)		SM+Coach^c^ (n=18^b^)		AC^d^ (n=18^b^)
Age (years), mean (SD)		40.11 (10.65)		47.5 (14.26)		49.55 (14.74)
Male, n (%)		13 (76)		14 (78)		11 (61)
White, n (%)		17 (100)		17 (94)		17 (94)
Hispanic Latino n (%)		2 (12)		1 (6)		1 (6)
**MARS^e^ total score^f^, mean (SD)**						
	Baseline	3.41 (0.46)	2.55 (0.45)		1.32 (0.45)	
	6 wk	2.66 (0.48)	2.16 (0.47)		1.44 (0.45)	
	12 wk	2.58 (0.48)	2.57 (0.48)		1.56 (0.45)	
**MARS results mixed effects model with group × time interaction**						
	Estimate (SE)	−0.85 (0.64)	2.08 (0.64)		—^g^	
	*t* test (*df*)	−1.32 (80)	−3.21 (80)		—	
	*P* value	.19	.002		—	
	Effect size change over time compared with AC	0.09	0.85		—	
**PHQ-9^h^ mean total score^i^, mean (SD)**						
	Baseline	15.41 (1.43)	12 (1.38)		13.27 (1.38)	
	6 wk	13.34 (1.55)	9.29 (1.50)		11.38 (1.38)	
	12 wk	12.2 (1.58)	8.7 (1.56)		11.45 (1.4)	
**PHQ-9 results mixed effects model with group × time interaction**						
	Estimate (SE)	−3.41 (1.99)	−2.13 (1.99)		—	
	*t* test (*df*)	1.71 (80)	−1.07 (80)		—	
	*P* value	.09	.28		—	
	Effect size change over time compared with AC	0.32	0.24		—	
**FIBSER^j^ mean total score^k^, mean (SD)**						
	Baseline	6.05 (1.00)	3.55 (0.97)		3.38 (0.97)	
	6 wk	3.83 (1.11)	1.51 (1.07)		4.88 (0.97)	
	12 wk	4.06 (1.11)	2.53 (1.14)		3.73 (1.01)	
**FIBSER results mixed effects model with group × time interaction**						
	Estimate (SE)	2.50 (1.39)	−2.66 (1.39)		—	
	*t* test (*df*)	−1.79 (80)	−1.91 (80)		—	
	*P* value	.07	.06		—	
	Effect size change over time compared with AC	0.40	0.54		—	

^a^SM-Only: secure messaging only.

^b^n=those enrolled into each arm. Outcomes analysis calculated using discontinued n, as reported in the *Results* section (SM-Only=13; secure messaging with coaching=14; attention control=18).

^c^SM+Coach: secure messaging with coaching.

^d^AC: attention control.

^e^MARS: Medication Adherence Rating Scale.

^f^The MARS has a scoring range of 1 to 10, with higher scores indicating higher adherence.

^g^Comparator.

^h^PHQ-9: 9-Item Patient Health Questionnaire.

^i^PHQ-9 has a scoring range of 0 to 27, with a higher score indicating a higher severity of symptoms.

^j^FIBSER: Frequency, Intensity, and Burden of Side Effects Ratings.

^k^Each of the FIBSER items range from 0 to 6, with the range for the 3 items summated ranging from 0 to 18. The summarized results have been reported.

### Change in Medication Adherence, Depressive Symptom Severity, and Side Effect Burden

Table 2 presents descriptions of the Veteran participants in each of the 3 arms, as well as results on the 3 key outcomes of this study. Change from baseline to 12 weeks on the MARS (increase=poor adherence) declined in the 2 intervention arms, SM-Only (ES=0.09; *P*=.19) or SM+Coach (ES=0.85; *P*=.002), whereas poor adherence increased for those in the AC arm. These differences only reached statistical significance for the comparison between the SM+Coach intervention compared with the AC.

Depression scores on the 9-Item Patient Health Questionnaire decreased for all 3 arms, although the decline was slightly larger for the SM-Only (ES=0.32; *P*=.09) and the SM+Coach (ES=0.24; *P*=.28) arms than for the AC arm. None of these differences in depression changes between the groups reached statistical significance. The 2 intervention arms indicated a decrease in side effects on the Frequency, Intensity, and Burden of Side Effects Ratings, whereas the side effect assessment for the AC arm increased. These differences were not statistically significant but indicated moderate ES (SM-Only vs AC: ES=0.40; *P*=.07; SM+Coach: ES=0.54; *P*=.06)

### Engagement With the Mobile App

In week 1, all veterans participants taking part in 1 of the 2 active treatment arms responded to at least one text. By week 6, 86% (30/35) had responded to at least one text, and in the final week, 80% (28/35) had responded to Annie at least once. The maximum number of days a participant could respond to a text was 5 days per week. In week 1, the average number of days responded across both treatment arms was 4.74 (SD 0.70). By week 6, this gradually declined to an average of 4.11 (SD 1.76) days. At week 12, Veteran participants responded to a text for an average of 3.37 (SD 2.02) days.

Table 3 presents Annie’s quotes and tips ordered from most helpful to least helpful. These scores were based on responses to the question, “How helpful did you find this?” (1 for “helpful,” 0 for “Not Helpful”). Although there was some variability in responses from veteran participants, most of the Annie’s quotes and tips were seen as helpful. The most helpful were those offering emotional support and promoting perseverance such as, “Every day may not be good...but there is something good in every day.” The lower-ranked quotes and tips tended toward concrete reminders about illness management such as “Do not adjust your medications on your own even if you are feeling better.”

**Table 3:** How helpful veterans perceived each Annie quote or tip.

Quote or tip	Value, mean (SD)^a^
Annie here with a quote of the day! “Every day may not be good...but there is something good in every day.” -Alice Morse Earl	64.44 (36.95)
Hi there, Annie here. Remember to be kind to yourself today. You are taking important steps to help yourself.	63.68 (35.91)
If you are having difficulties with your prescription, make sure to call or send a secure message to your VA care team. -Annie	63.43 (35.84)
Annie here with a quote of the day! “Our greatest glory is not in never falling, but in rising every time we fall.” -Confucius	62.07 (36.8)
Annie here with a quote of the day! “Tough times don’t last, tough people do”—Robert Schuller	60.85 (37)
Annie here, take note if your mood improves after you do the behavior you set as a goal.	60.40 (36.42)
Make plans to act to help your mood, such as talking to a friend or spending time with your pet. -Annie	57.62 (36.43)
Hi there, Annie here. The path to getting better sometimes has ups and downs.	57.57 (41.68)
Sometimes progress has ups and downs. Take care, Annie.	57.52 (33.08)
Annie here with a quote of the day! “I will be stronger than my sadness.” -Jasmine Wargi	57.42 (37.79)
Annie here with a quote of the day! “Have patience with all things, but first of all with yourself.” -Saint Francis de Sales	57.42 (37.46)
Annie here with a reminder to do the behavior you set as a goal in this study.	56.36 (37.44)
Be sure to put aside time to work on your behavior goal. Thanks Annie	55.6 (39.48)
Try to be patient. Progress can take time. Thanks, Annie.	51.31 (39.87)
Do not adjust your medications on your own even if you are feeling better. -Annie	50 (40.71)

^a^Possible range from 0 to 100 with 0 indicating veterans never found this quote helpful, to 100 where veterans always found the quote helpful.

## Discussion

### Principal Findings

This pilot study comparing 2 forms of a mobile health intervention to an AC condition demonstrated feasibility, acceptability, and small to moderate ES in improving antidepressant adherence and reducing side effect burden. It did not appear to reduce depressive symptom severity. Consistent with previous research, the intervention that included brief synchronic meetings with a coach appeared to have greater benefit than SMS alone. In a recent review of digital psychiatry, Torous et al [23] recommended that health care systems support digital navigators that assist both clinicians and patients in adopting mobile technologies to manage their health. Such navigators, akin to the coaches in our study, can promote ongoing engagement with the application so that the patient may eventually benefit from the technology.

Veteran participants who remained in the study engaged with the mobile app over 12 weeks in contrast to the prior studies. Moreover, 80% of the participants remained engaged with Annie at weeks 6 and 12 of the intervention. Prior estimates of patient engagement for mobile apps report lower retention on average [24] than what was observed in this study. Our retention was comparable with retention found for a mobile app targeting self-management of schizophrenia [11] and slightly higher than what was promoted in a study focusing specifically on people with depression, which found that app engagement dropped off significantly after 2 weeks [25]. The intervention designed herein combined secure messaging with some intermittent contact that may boost retention.

In addition to the coaching, our sustained retention may be because of the direct integration of Annie into the patient’s current treatment, and app results were shared with both Veteran participant and their provider. The actual content of the app was informed by the specific clinical goal at hand: encouraging veterans to remain on their antidepressant long enough to experience and evaluate its true effectiveness. The intervention also included tips and quotes intended to be supportive or inspirational and Veteran participants rated the degree to which these were helpful. It appears that those who provided general encouragement were rated as more helpful than those who recommended specific self-care behaviors. A similar patient engagement strategy using a chatbot with sophisticated branching logic was tested among individuals with eating disorders [26]. As observed in our study, these chatbot users rated the personalized messaging highly and remained engaged with the technology over time.

Simon et al [27] conducted a web messaging program to support depression follow-up care that combined algorithm-determined responses with real-time text communication with a care manager when required. The care manager then consulted with a physician about treatment options and responses to patient communications. Similar to our study, this program yielded improved antidepressant adherence but also achieved a greater reduction in depressive symptoms over 5 months.

The differential dropout between the 2 interventions and control condition suggests that a significant portion of these Veteran participants did not accept the Annie intervention. Consistent with prior research on mobile apps in mental health, dropout can be high and often occurs within the first month of an intervention. This limits our ability to estimate the true impact across the entire target population of veterans initiating antidepressants. Future research is needed to better understand these technology refusers and to determine whether there are strategies to enhance their experience and retention.

Annie was harnessed in the VA’s response to the COVID-19 pandemic [28]. The algorithm-derived messaging protocol assessed how veterans were feeling and their temperature and probed for difficulty breathing. Referrals to contact VA providers occurred based on Veteran responses. Program evaluation revealed that this likely reduced the overall cost of COVID-19 management as it supplanted direct contact with the VA. Moreover, 75% of the respondents to a postintervention survey indicated that Annie’s messages were helpful. In light of these successes, VA continues to explore how algorithm-based text messaging integrated into the care provided by a large health care system can improve population health in a cost-effective manner.

### Limitations

This study has several limitations. It was a pilot study designed to demonstrate feasibility and determine ES but was not powered to perform definitive tests of statistical significance between treatment arms. Moreover, there was a greater dropout in the active intervention arms than in the AC arm. It is not clear why this occurred, but it is possible that those with negative experiences around texting stopped study participation, thereby biasing estimates of positive impact and overall estimates of patient engagement over 12 weeks.

### Conclusions

This study presents an SMS protocol developed specifically to address key barriers to antidepressant adherence. Consistent with the overarching recommendation to integrate technology into clinical contexts and practice, the program was developed to collect information demonstrated to undermine antidepressant adherence such as perceived effectiveness and side effect burden. SMS responses aimed to educate Veteran participants based on their responses to the probes. In addition, this information was presented in a graphic format and was shared with both the patients and their providers. Veterans engaged with Annie and there is preliminary support that this enhanced their self-management of depression, although differential impact on actual depressive symptom burden did not occur. However, there was differential dropout with significantly greater rates in the interventions that clouds the interpretation of results.

## Appendix

Multimedia Appendix 1 CONSORT-eHEALTH checklist (V 1.6.1).

## Abbreviations

AC: attention control
ES: effect size
MARS: Medication Adherence Rating Scale
MHV: My HealtheVet
SM+Coach: secure messaging with coaching
SM-Only: secure messaging only
VA: Veterans Affairs

## Data Availability

As these data were collected within the Department of VA, they will not be made available.
